# Iron-Regulated Phospholipase C Activity Contributes to the Cytolytic Activity and Virulence of *Acinetobacter baumannii*

**DOI:** 10.1371/journal.pone.0167068

**Published:** 2016-11-22

**Authors:** Steven E. Fiester, Brock A. Arivett, Robert E. Schmidt, Amber C. Beckett, Tomislav Ticak, Mary V. Carrier, Rajarshi Ghosh, Emily J. Ohneck, Maeva L. Metz, Marlo K. Sellin Jeffries, Luis A. Actis

**Affiliations:** 1 Department of Microbiology, Miami University, Oxford, Ohio, United States of America; 2 Biology Department, Middle Tennessee State University, Murfreesboro, Tennessee, United States of America; East Carolina University Brody School of Medicine, UNITED STATES

## Abstract

*Acinetobacter baumannii* is an opportunistic Gram-negative pathogen that causes a wide range of infections including pneumonia, septicemia, necrotizing fasciitis and severe wound and urinary tract infections. Analysis of *A*. *baumannii* representative strains grown in Chelex 100-treated medium for hemolytic activity demonstrated that this pathogen is increasingly hemolytic to sheep, human and horse erythrocytes, which interestingly contain increasing amounts of phosphatidylcholine in their membranes. Bioinformatic, genetic and functional analyses of 19 *A*. *baumannii* isolates showed that the genomes of each strain contained two phosphatidylcholine-specific phospholipase C (PC-PLC) genes, which were named *plc1* and *plc2*. Accordingly, all of these strains were significantly hemolytic to horse erythrocytes and their culture supernatants tested positive for PC-PLC activity. Further analyses showed that the transcriptional expression of *plc1* and *plc2* and the production of phospholipase and thus hemolytic activity increased when bacteria were cultured under iron-chelation as compared to iron-rich conditions. Testing of the *A*. *baumannii* ATCC 19606^T^ *plc1*::*aph-FRT* and *plc2*::*aph* isogenic insertion derivatives showed that these mutants had a significantly reduced PC-PLC activity as compared to the parental strain, while testing of *plc1*::*ermAM/plc2*::*aph* demonstrated that this double PC-PLC isogenic mutant expressed significantly reduced cytolytic and hemolytic activity. Interestingly, only *plc1* was shown to contribute significantly to *A*. *baumannii* virulence using the *Galleria mellonella* infection model. Taken together, our data demonstrate that both PLC1 and PLC2, which have diverged from a common ancestor, play a concerted role in hemolytic and cytolytic activities; although PLC1 seems to play a more critical role in the virulence of *A*. *baumannii* when tested in an invertebrate model. These activities would provide access to intracellular iron stores this pathogen could use during growth in the infected host.

## Introduction

*Acinetobacter baumannii* is a Gram-negative coccobacillus pathogen linked to severe nosocomial infections including pneumonia, bacteremia, urinary tract infections and necrotizing fasciitis [1, 2]. *A*. *baumannii* infections have been commonly associated with immunocompromised patients; however, cases of community-acquired *A*. *baumannii* infections in healthy individuals have also been reported [3]. Reports have also associated *A*. *baumannii* with wound infections acquired by combatants deployed to Iraq earning it the popularized name ‘Iraqibacter’ [4].

Treatment of *A*. *baumannii* infections is exceedingly difficult due to increasing multi-drug resistance and the limited understanding of its virulence factors, conditions that have a paramount impact on human health worldwide. While the mechanisms of antibiotic resistance associated with this emerging pathogen have been extensively studied, there is a troublesome paucity of literature reporting the molecular mechanisms of virulence associated with *A*. *baumannii* pathogenicity [5]. Among the more understood properties that make *A*. *baumannii* a successful pathogen is its versatility in acquiring iron [6].

The majority of iron in a host is intracellular; thus the availability of intracellular iron-containing molecules such as hemin, hemoglobin and ferritin is dependent on the lysis of host cells and their subsequent release due to cell and tissue damage found in wounds [7, 8]. The liberation of intracellular nutrients may be accomplished by bacterial-mediated cell damage such as that described in *V*. *cholerae* infections, in which hemolysin-based cytotoxicity lyses intestinal epithelial cells and erythrocytes releasing intracellular iron compounds into the extracellular environment for bacterial utilization [9]. One avenue by which bacterial pathogens can lyse host cells is by producing phospholipases, which act on phospholipids in host membranes resulting in membrane destabilizing products thereby leading to cytolysis and the release of host intracellular contents [10].

The *A*. *baumannii* ATCC 19606^T^ strain genome contains genes encoding proteins harboring phospholipase domains including four with a patatin-like protein (PLP) phospholipase domain, one outer membrane protein with a phospholipase A1 domain and two with a phospholipase C domain (http://www.broadinstitute.org/). A more recent report showed that the genome of this strain also includes three genes the products of which are proteins that harbor PLD domains [11]. These phospholipases differ in the types of reactions they catalyze; PLP phospholipases are non-specific acyl lipid hydrolases that cleave the acyl ester bond of a phospholipid [12], phospholipase A1 specifically cleaves phospholipids through the hydrolysis of the fatty acyl ester bond at the *sn*-1 position of the glycerol moiety [13], and phospholipase C and phospholipase D cleave before and after the phosphate, respectively.

Patatins are plant storage glycoproteins with lipid acyl hydrolase activity that account for 30–40% of the total soluble proteins in potatoes [14]. The first, and one of the few, PLPs to be characterized in bacteria was the ExoU protein from *Pseudomonas aeruginosa*, which was shown to have phospholipase activity [15, 16]. While bacterial PLPs have not been linked to cytolysis, their presence in the genomes of animal and plant pathogens/symbionts is significantly higher than in the genomes of non-pathogens [15]. The bacterial phospholipase A1 (PhlA) from *Serratia marcescens* has been implicated in hemolysis of human erythrocytes and cytotoxicity to cervical cancer HeLa and 5637 human bladder epithelial cells [17]. The phospholipase C of *Clostridium perfringens*, which is also known as the α toxin, causes cytolysis, tissue destruction and necrosis [10]. The phospholipase C produced by *P*. *aeruginosa* has been linked to hemolysis, tissue destruction and pathologies reminiscent of burn infections [10]. Purified phospholipase D, such as that produced by *Corynebacterium pseudotuberculosis*, is dermonecrotic and fatal when injected into animals [18]. While many of the phospholipases encoded within the *A*. *baumannii* ATCC 19606^T^ genome have possible implications in cytolysis and the ultimate release of iron-rich intracellular contents, the roles of only a few of these phospholipases have been elucidated in this pathogen. Specifically, the role of a phospholipase C and a phospholipase D has been associated with cytolytic activity to the FaDu hypopharyngeal carcinoma epithelial cell line [19] and serum survival and invasion into both human bronchial epithelial BEAS-2B cells and HeLa cells, respectively [20]. A recent report showed that three phospholipase D proteins play a critical role in the pathobiology of the ATCC 19606^T^ strain [11].

Taken together, these observations indicate that bacterial pathogens can gain access to additional intracellular iron pools and other nutrients present in erythrocytes and tissues through the expression of hemolytic/cytolytic activities. Supernatants of *A*. *baumannii* cultures grown under iron-chelation are hemolytic to horse erythrocytes and possess phospholipase C activity [21, 22]. Our report describes the characterization of *plc1* and *plc2* and the involvement of the protein products of these two genes in the hemolytic, cytolytic and virulence phenotypes displayed by the *A*. *baumannii* ATCC 19606^T^ strain and isogenic derivatives affected in the expression of these two genes.

## Materials and Methods

### Bacterial strains, plasmids, media and culture conditions

The bacterial strains and plasmids used in this work are listed in Table 1. All bacterial strains were routinely stored as Luria-Bertani (LB) broth/glycerol stocks at -80°C [23]. *Escherichia coli* DH5α recombinant clones were cultured in LB broth or on LB agar (LBA) [23] supplemented with appropriate antibiotics and incubated overnight (12–14 h) at 37°C. *A*. *baumannii* strains as well as the *E*. *coli* MG1655 strain were subcultured from LBA into Chelex 100-treated trypticase soy broth dialysate (TSBD) [24] and grown for 24 h at 37°C with shaking at 200 rpm. These cultures were then used to inoculate fresh TSBD or TSBD containing 10% erythrocytes at a 1/100 ratio and grown for 24 h at 37°C with shaking at 200 rpm unless otherwise indicated. Bacterial cells were enumerated after 24 h using flow cytometry. Culture medium supplemented with erythrocytes was prepared by centrifuging whole blood at 1,000 *x g*, resuspending and washing the erythrocyte pellet three times in erythrocyte wash buffer (20 mM KH_2_PO_4_, 60 mM Na_2_HPO_4_ and 120 mM NaCl, pH 8.0) [9] and then resuspending the pellet to a final erythrocyte concentration of 10% in TSBD. Iron-repleted culture conditions were accomplished through the addition of 50 μM FeCl_3_ dissolved in 0.01 N HCl, while iron-chelated conditions were generated by treating TSB with Chelex 100 (Bio-Rad Laboratories). Bacterial growth curves were determined in octuplet using 96-well microtiter plates containing TSBD under the aforementioned culturing conditions over a 24-h time period. OD_600_ values of these cultures were recorded hourly.

**Table 1:** Strains and Plasmids Used in This Study.

Strain/plasmid	Relevant characteristic(s)^*a*^	Source/reference
**Strains**		
***A*. *baumannii***		
17978	Clinical isolate	ATCC
19606^T^	Clinical isolate, type strain	ATCC
19606^T^ 3452	*plc1*::*aph-FRT* derivative of 19606^T^; Km^R^	This work
19606^T^ 3452.C	3452 derivative harboring pMU1079; Km^R^, Amp^R^	This work
19606^T^ 3452.E	3452 derivative harboring pWH1266; Km^R^, Amp^R^, Tet^R^	This work
	*plc2*::*aph* derivative of 19606^T^; Km^R^	
19606^T^ 3430		This work
19606^T^ 3430.C	3430 derivative harboring pMU1080; Km^R^, Amp^R^	This work
19606^T^ 3430.E	3430 derivative harboring pWH1266; Km^R^, Amp^R^, Tet^R^	This work
19606^T^ 3494	*plc1*::*ermAM/plc2*::*aph* derivative of 19606^T^; Em^R^, Km^R^	This work
AB3340	Wound isolate	Zurawski, D.
AB3560	Wound isolate	Zurawski, D.
AB3638	Wound isolate	Zurawski, D.
AB3806	Wound isolate	Zurawski, D.
AB4026	Wound isolate	Zurawski, D.
AB4052	Wound isolate	Zurawski, D.
AB4456	Wound isolate	Zurawski, D.
AB4498	Wound isolate	Zurawski, D.
AB5075	Wound isolate	Zurawski, D.
AB5197	Wound isolate	Zurawski, D.
AYE	Wound isolate	ATCC
LUH 5875	Clinical isolate, reference strain, EU clone III	[25]
LUH 07672	Clinical isolate, EU clone III	[26]
LUH 8809	Clinical isolate, EU clone I	[27]
LUH 13000	Clinical isolate, EU clone II	Dijkshoorn, L.
RUH 134	Clinical isolate, reference strain, EU clone II	[28]
RUH 875	Clinical isolate, reference strain, EU clone I	[28]
***E*. *coli***		
DH5*α*	Used for recombinant DNA methods	Gibco-BRL
MG1655	Displays γ-hemolysis	Blattner, F. R.
**Plasmids**		
pCR8/GW/TOPO	PCR cloning vector; Sp^R^	Life Technologies
pKD13	Source of *aph-FRT* cassette; Ap^R^, Km^R^	Crosa, J. H.
pUC4K	Source of *aph* cassette; Ap^R^, Km^R^	Life Technologies
pIL252	Source of *ermAM* cassette; Em^R^	Kruse, T.
pEX100T	Mobilizable suicide plasmid in 19606^T^; Ap^R^	ATCC
pCR-Blunt	PCR cloning vector; Km^R^, Zeo^R^	Life Technologies
pWH1266	*A*. *baumannii-E*. *coli* shuttle vector; Amp^R^, Tet^R^	[29]
pMU1039	pCR8/GW/TOPO harboring *plc2*; Sp^R^	This work
pMU1040	Insertion of *aph* into pMU1039; Sp^R^, Km^R^	This work
pMU1042	pCR8/GW/TOPO harboring *plc1*; Sp^R^	This work
pMU1073	pCR-Blunt harboring *plc1*; Km^R^, Zeo^R^	This work
pMU1074	pCR-Blunt harboring *plc2*; Km^R^, Zeo^R^	This work
pMU1076	Insertion of pMU1040 into pEX100T; Ap^R^, Km^R^	This work
pMU1079	pWH1266 harboring *plc1*; Amp^R^, Tet^R^	This work
pMU1080	pWH1266 harboring *plc2*; Amp^R^, Tet^R^	This work
pMU1089	Insertion of *aph-FRT* into pMU1042; Sp^R^, Km^R^	This work
pMU1091	Insertion of pMU1089 into pEX100T; Ap^R^, Km^R^	This work
pMU1101	Insertion of *ermAM* into pMU1042; Sp^R^, Em^R^	This work
pMU1108	Insertion of pMU1101 into pEX100T; Ap^R^, Em^R^	This work

^*a*^Ap^R^, ampicillin resistance; Em^R^, erythromycin resistance; Km^R^, kanamycin resistance; Sp^R^, spectinomycin resistance; Tet^R^, tetracycline resistance; Zeo^R^, Zeocin resistance.

Defibrinated sheep and horse erythrocytes were obtained from Cleveland Scientific, Ltd., and sodium citrate-treated whole human blood was purchased from Bioreclamation, LLC. A549 human alveolar epithelial cells were passaged three times in Dulbecco’s modified Eagle’s medium (DMEM) supplemented with 10% heat-inactivated fetal bovine serum, 100 IU penicillin, and 100 μg/ml streptomycin at 37°C in the presence of 5% CO_2_. Approximately 1x10^5^ A549 cells, as enumerated using a hemocytometer, were seeded into each well of a 96-well, white, opaque tissue culture plate for a fourth passage that was incubated at 37°C in the presence of 5% CO_2_ for 24 h without antibiotics. A549 cell monolayers were infected with 10^6^ bacteria suspended in DMEM and incubated at 37°C in the presence of 5% CO_2_ for 24 h. The cell monolayers were washed three times with DMEM prior to performing cytolysis assays.

### General DNA procedures

**Fig 1:**
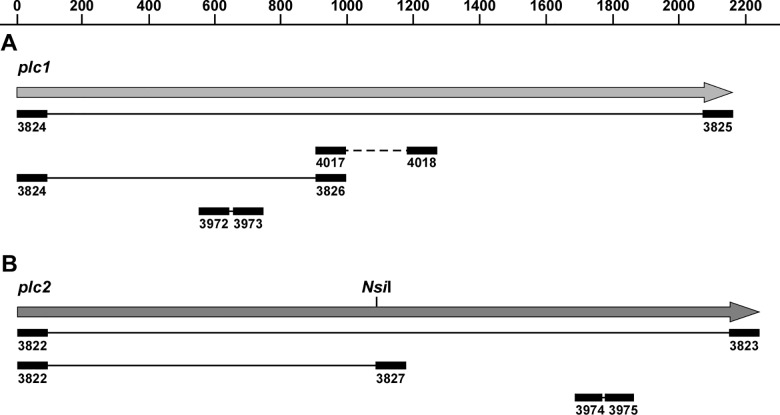
*A*. *baumannii* ATCC 19606^T^ genetic loci harboring the monocistronic *plc1* and *plc2*. Genetic map of *plc1* (A) and *plc2* (B). The horizontal arrows represent the direction of transcription of predicted coding regions. Numbers on top of A indicate size in base pairs. The location of the *Nsi*I restriction site used to generate the *plc2*::*aph* insertion derivative is indicated in panel B. Black rectangles connected with solid black lines identify primers and amplicons used to clone *plc1* and *plc2*, test their presence in different *A*. *baumannii* strains and examine their transcription by qRT-PCR. Numbers underneath of each black rectangle indicate primer numbers as listed in S1 Table. The black rectangles connected with a dashed line in panel A indicate the location of the primers 4017 and 4018 and the deleted fragment replaced with the *aph-FRT* gene used to generate the *plc1* deletion/insertion derivative by inverse PCR.

Total genomic DNA was isolated using an adapted mini-scale procedure from a previously published method [30], and plasmid DNA was isolated using commercial kits (Qiagen). Restriction digests were performed as suggested by the supplier (New England Biolabs) and size-fractionated by agarose gel electrophoresis [23]. PCR primer pairs 3824/3826 and 3822/3827 (S1 Table), which hybridize internally of *plc1* and *plc2*, respectively (Fig 1), were used to confirm the presence of these two genes in 19 *A*. *baumannii* clinical isolates.

### Sequence acquisition and phylogenetic analyses

Nucleotide sequences were analyzed with DNASTAR (DNASTAR, Inc.), BLAST [31] and data available from the Broad Institute. *In silico* identification of potential ferric uptake repressor (Fur)-binding sites upstream of *plc1* and *plc2* was performed using a small training set of predicted Fur-binding sites [32], which were analyzed using MEME Suite 4.10.0 [33]. GLAM2 PSSM was used to generate a WebLogo representing the *A*. *baumannii* Fur-binding motif, and MAST was used to search nucleotide sequences upstream of *plc1* and *plc2* [34–37]. The PLC1 and PLC2 amino acid sequences from *A*. *baumannii* ATCC 19606^T^ were used as queries for BLASTp [31] to obtain similar sequences, excluding additional amino acid sequences from *A*. *baumannii*. A total of 101 amino acid sequences were retrieved by setting an arbitrary cutoff of approximately 40% amino acid identity for phylogenetic comparisons to PLC1, PLC2, and the hemolytic (PLCH) and non-hemolytic (PLCN) *P*. *aeruginosa* phospholipase C proteins (GI 489205171 and GI 489204069), which were added manually. These sequences, which were aligned with MUSCLE using default settings, were analyzed with the multiple sequence alignment (MSA) MEGA6 software package [38, 39]. The MSA analysis was done using an apparent-maximum-likelihood method encompassing the WAG (Whelan Goldman) model [40] with a discrete Gamma distribution and rate calculations among invariable sites with FastTree [41]. The phylogenetic tree, which was drawn to scale with the highest log likelihood (-714043.274), represents 109 residues analyzed with 1,099 positions in the final dataset.

### Site-directed insertional mutagenesis of *plc* genes

The 2.2-kb *plc1* gene (A1S_0043 following the *A*. *baumannii* ATCC 17978 genome annotation) was amplified from ATCC 19606^T^ genomic DNA with *Taq* DNA polymerase and primers 3824 and 3825 (S1 Table and Fig 1). The *plc1* amplicon was cloned into pCR8/GW/TOPO generating pMU1042 (Table 1). Inverse PCR was performed using pMU1042 as a template, Phusion DNA polymerase (New England Biolabs) and primers 4017 and 4018 (S1 Table), which hybridize within the *plc1* gene (Fig 1). The *aph-FRT* cassette, which codes for kanamycin resistance (Km^R^), was amplified from pKD13 with Phusion DNA polymerase and primers 4003 and 4004 (S1 Table) and ligated within *plc1* using the inverse PCR amplicon described above to generate pMU1089 (Table 1). The *ermAM* cassette, which codes for erythromycin resistance (Em^R^), was amplified from pIL252 (Table 1) with Phusion DNA polymerase and primers 4046 and 4047 (S1 Table) and ligated within the *plc1* coding region using the inverse PCR amplicon described above to generate pMU1101 (Table 1). Phusion DNA polymerase and primers 3824 and 3825 (S1 Table) were used to amplify *plc1*::*aph-FRT* and *plc1*:: *ermAM*, which were each subcloned into the *Sma*I site of pEX100T to generate pMU1091 and pMU1108, respectively (Table 1).

The 2.2-kb *plc2* gene (A1S_2055 following the *A*. *baumannii* ATCC 17978 genome annotation) was PCR amplified from ATCC 19606^T^ genomic DNA using *Taq* DNA polymerase and primers 3822 and 3823 (S1 Table and Fig 1), and the resulting amplicon was ligated into pCR8/GW/TOPO to generate pMU1039 (Table 1). Phusion DNA polymerase and primers 3171 and 3172 (S1 Table) were used to amplify the *aph* cassette from pUC4K (Table 1), which was inserted into the unique *Nsi*I site of the *plc2* gene (Fig 1) after end repair of *Nsi*I-digested pMU1039 (Table 1) with the End-It Kit (Epicentre) resulting in pMU1040 (Table 1). Phusion DNA polymerase and primers 3822 and 3823 (S1 Table) were used to amplify *plc2*::*aph*, which was subsequently cloned into the *Sma*I site of pEX100T to generate pMU1076 (Table 1).

Electrocompetent ATCC 19606^T^ cells were electroporated with pMU1091 and pMU1076 as described before [42] to generate the 3452 *plc1*::*aph-FRT* and 3430 *plc2*::*aph* isogenic derivatives, respectively (Table 1). For the generation of the 3494 *plc1*::*ermAM/plc2*::*aph* double insertion isogenic derivative, electrocompetent 3430 cells were electroporated with pMU1108 (Table 1). The ATCC 19606^T^ 3430 and 3452 isogenic derivatives were selected on LBA plates containing 40 μg/ml kanamycin, while the 3494 derivative was selected on LBA supplemented with 40 μg/ml erythromycin. All isogenic derivatives were plated on LBA plates supplemented with 10% sucrose to ensure loss of pMU1076, pMU1091 and pMU1108. Proper allelic exchanges were confirmed with PCR using external primers 3905 and 3906 (*plc1*) and 3815 and 3918 (*plc2*) (S1 Table).

### Genetic complementation of the *plc1* and *plc2* isogenic derivatives

The *A*. *baumannii* ATCC 19606^T^ *plc1* and *plc2* genes were PCR amplified from ATCC 19606^T^ genomic DNA using Phusion DNA polymerase and primers 3894 and 3895 (*plc1*) and 3892 and 3893 (*plc2*), all of which included *Bam*HI restriction sites (S1 Table). The respective *plc1* and *plc2* amplicons were ligated into pCR-Blunt resulting in pMU1073 and pMU1074 (Table 1). Both pMU1073 and pMU1074 were digested with *Bam*HI and the *plc1* and *plc2* fragments were each subcloned into the cognate *Bam*HI site of the *E*. *coli-A*. *baumannii* shuttle vector pWH1266 generating pMU1079 and pMU1080 (Table 1).

Electrocompetent 3452 (*plc1*::*aph-FRT*) and 3430 (*plc2*::*aph*) cells were electroporated with the empty shuttle vector pWH1266 resulting in the 3452.E and 3430.E transformants, respectively, which were selected on LB agar supplemented with 1 mg/ml of ampicillin. Electrocompetent 3452 (*plc1*::*aph-FRT*) and 3430 (*plc2*::*aph*) cells were also electroporated with pMU1079 and pMU1080, respectively, and the 3452 and 3430 complemented derivatives (3452.C and 3430.C) were selected on LB agar supplemented with 1 mg/ml of ampicillin. The presence of pMU1079 and pMU1080 was confirmed by restriction analysis of plasmid DNA recovered from transformants grown in LB containing 150 μg/ml of ampicillin.

### Transcriptional analyses

Bacterial strains were each grown as five independent 1-ml cultures for 24 h in TSBD or TSBD supplemented with 50 μM FeCl_3_ at 37°C with shaking at 200 rpm. The 24-h time point was chosen for RNA isolation because there was not apparent hemolytic activity until this time. RNA isolation, cDNA synthesis and qRT-PCR analyses were performed as previously described [43]. Briefly, bacterial cells were lysed in lysis buffer [0.3 M sodium acetate (pH 4.0), 30 mM EDTA and 3% SDS] previous to RNA purification following the manufacturer’s protocol included with the Maxwell 16 LEV simplyRNA Tissue Kit (Promega). Total RNA concentrations and the OD_260/280_ ratios of each RNA sample were assessed using a NanoDrop 2000 UV-Vis spectrophotometer (Thermo Fisher Scientific). RNA integrity was assessed using a RNA 6000 NanoKit for the Bioanalyzer 2100 (Agilent Technologies) and the manufacturers’ protocols. Only RNA samples with OD_260/280_ ratios > 1.7 and RNA integrity numbers (RINs) > 5 were further processed for qRT-PCR analysis. The iScript cDNA synthesis kit (Bio-Rad Laboratories) was utilized for cDNA synthesis from 100 ng of total RNA template following the manufacturer’s protocol, and iQ SYBR Green (Bio-Rad Laboratories) was used to examine gene transcription following the manufacturer’s recommendations. The 10-μl reaction mix included 0.4 μl cDNA, 5 μl iQ SYBR-Green supermix and 300 nM of forward and reverse primers.

Primers 3966 and 3967 (S1 Table) were used to amplify a 179-bp internal fragment of the 16S ribosomal RNA gene, which served as an internal control of gene expression, while primers 3970 and 3971 (S1 Table) were used to amplify a 156-bp internal fragment of *bauA*, which served as a positive control for gene expression under iron chelation. Primers 3972 and 3973 or 3974 and 3975 (S1 Table and Fig 1) were used to amplify a 197-bp internal fragment of *plc1* and a 181-bp internal fragment of *plc2*, respectively. The cycling conditions for qRT-PCR assays, which were performed on a Bio-Rad CFX Connect real-time PCR detection system, were as follows: 95°C for 3 min followed by 40 cycles of 95°C for 10 s and 60°C for 45 s. Relative expression of *plc1* and *plc2* between iron-chelated and iron-repleted conditions was quantified by the standard curve method in which serial dilutions of cDNA samples served as standards. Samples containing no cDNA template were used as negative controls. qPCR efficiencies were as follows: 16S, 82.4%; *bauA*, 100.9%; *plc1*, 106.7%; and *plc2*, 97.7%. All samples were analyzed in triplicate and melting curve data were included in the analysis to confirm primers specificity. Data analysis also showed that there were no significant differences in 16S expression between iron-chelated and iron-repleted conditions. Therefore, the expression of *plc1* and *plc2* was normalized to that of the 16S ribosomal RNA gene.

### Cell-type phospholipid content

The Bligh-Dyer method was utilized to extract total lipids from sheep, human, and horse erythrocytes, A549 pneumocytes, and *G*. *mellonella* [44]. Erythrocytes were extracted by the addition of 2:0.8:2 parts of methanol, water and chloroform. A549 cells were processed with 2:1.8:2 parts of methanol, water and chloroform. *G*. *mellonella* larvae were homogenized and extracted with 2:0.8:2 parts methanol, water, and chloroform [45]. The lipid-containing chloroform fraction was analyzed by normal-phase HPLC. Phosphatidylcholine and phosphatidylethanolamine levels in the samples were calculated using an Ultimate 3000 high performance liquid chromatography system (Thermo, Germering, Germany) coupled with a Corona charged aerosol detector instrument (Thermo, Chelmsford, MA, USA) [46]. Chromatographic separation was carried out using a Phenomenox Silica (150 x 4.6 mm; 3μ) column at a flow rate of 1 ml/min. The mobile phase consisted of (A) butyl acetate/methanol (4:1) and (B) butyl acetate/methanol/water (1:3:1) with the following gradient elution: 0%-100% B at 0–15 min, 100% B at 15–17 min, 100%-0% B at 17–21 min, and then equilibrated with 100% A for 4 min. The column temperature was set at 50°C and the injection volume was 10 μl. The nitrogen gas pressure and response range of the detector was set at 35 psi and 500 pA, respectively. The Chromeleon 6.8 software was used for data processing. Identification of isolated compounds was based on retention times of authentic standards.

### Phospholipase C and cytolysis assays

The presence of phosphatidylcholine-specific phospholipase C activity in *A*. *baumannii* culture supernatants obtained after centrifugation at 15,000 *x g* for 30 min was tested with the Amplex Red PC-PLC assay kit (Molecular Probes) using lecithin as a substrate and following the conditions suggested by the manufacturer’s protocol. Erythrocytes incubated in the presence of bacteria were diluted 1:1000 into filter-sterilized FACSFlow sheath fluid (BD Biosciences) for differential interference microscopy (DIC) and enumeration using flow cytometry. Erythrocyte morphological changes were observed in these samples using DIC microscopy on a Zeiss 710 Laser Scanning Confocal System (Carl Zeiss Microscopy GmbH). The number of erythrocytes present in each analyzed sample was quantified using a FACScan flow cytometer (BD Biosciences). Flow Cytometry Absolute Count Standard beads (Bangs Laboratories, Inc.) were added 1:40 to diluted samples to standardize volumes amongst 5-second samplings. Erythrocyte populations were gated using the forward and side scatter channels.

The relative number of A549 cells remaining in cell culture following incubation with *A*. *baumannii* strains was assessed using the CellTiter-Glo luminescent cell viability assay (Promega) following the manufacturer’s instructions. Briefly, the number of A549 cells remaining after incubation with bacteria was assessed by measuring the luminescence resulting from the reaction of the provided Ultra-Glo recombinant luciferase with ATP released from metabolically active A549 cells. The relative luminescence units (RLUs) produced, and thus the relative number of viable A549 cells remaining following infection, was quantified using a FilterMax F5 microplate reader (Beckman Coulter) and reported as a ratio of RLUs produced following lysis of infected A549 cells versus the RLUs produced following lysis of uninfected A549 cells.

### Galleria mellonella virulence assays

Virulence assays were conducted using the *G*. *mellonella* model as previously described [47]. Briefly, assays were performed by injecting in triplicate 10 randomly selected healthy final-instar *G*. *mellonella* larvae (n = 30) injected with 10^5^ CFUs/larva (± 0.5 log) of the ATCC 19606^T^ or its isogenic derivatives suspended in sterile phosphate-buffered saline (PBS). Non-injected larvae or larvae injected with five microliters of sterile PBS were included as controls. After injection, larvae were incubated in darkness at 37°C, and the numbers of dead larvae were assessed at 24-hour intervals over 5 days with removal of dead larvae at times of inspection. Trials were repeated if more than two deaths were observed in any of the control groups.

### Statistical analyses

The Student’s *t*-test or one-way analysis of variance (ANOVA), both provided as part of the GraphPad InStat software package (GraphPad Software, Inc.), were used to analyze the statistical significance of data, as appropriate for the data set. Means of experimental data were compared to the means of the respective control groups using the Tukey-Kramer multiple comparisons post-hoc test. Survival curves were plotted using the Kaplan-Meier method [48] and analyzed for statistical significance using the log-rank test of survival curves (SAS Institute Inc.). Significances for all data analyses were set *a priori* at *P* ≤ 0.05.

## Results

### Membrane lipid preference for *A*. *baumannii* hemolytic activity

**Fig 2:**
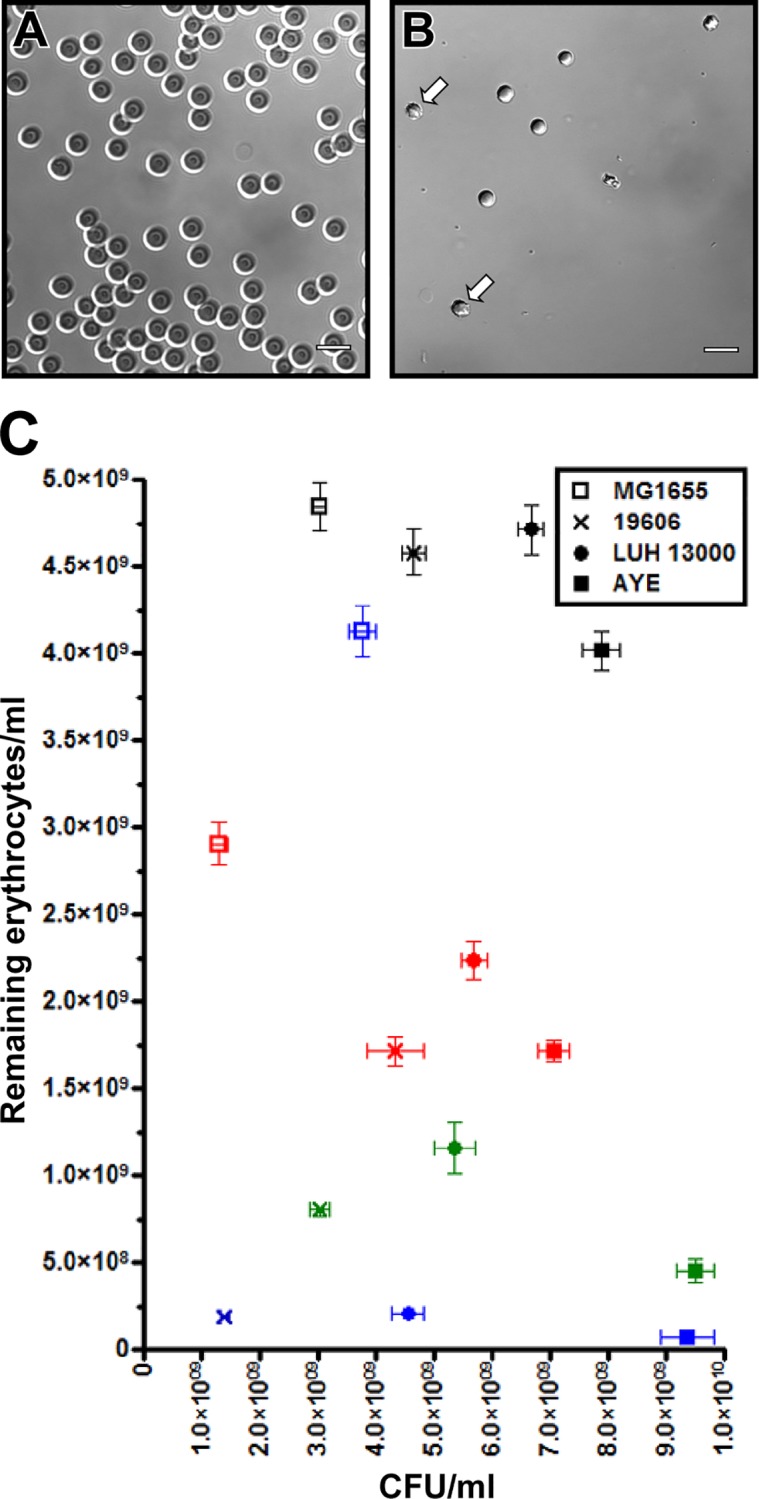
Hemolytic activity of *E*. *coli* and *A*. *baumannii*. DIC image of horse erythrocytes incubated in TSBD alone (A) or TSBD inoculated with ATCC 19606^T^ (B). White arrows identify damaged erythrocytes. White bars represent 10 μm. (C) Quantification of intact sheep (black symbols), human (red symbols) or horse (blue symbols) erythrocytes remaining after incubation with *E*. *coli* MG1655 or each of the three different *A*. *baumannii* strains. Horse erythrocytes were also enumerated after incubation with these three *A*. *baumannii* strains grown in iron-repleted TSBD (green symbols). All incubations were conducted for 24 h at 37°C with shaking at 200 rpm. Error bars represent the standard error (SE) of the mean for data collected in triplicate from three individual biological samples.

DIC microscopy of horse erythrocytes incubated in the presence of ATCC 19606^T^ cells shows that the presence of this strain significantly decreases the number of intact red blood cells remaining in TSBD culture after incubation at 37°C for 24 h (Fig 2, panels A and B). In addition, the horse erythrocytes showed morphological changes characteristic of cell membrane damage following incubation with ATCC 19606^T^ (Fig 2B). In contrast, the number and morphology of sheep erythrocytes did not change after co-incubation under the same conditions (S1 Fig). These data prompted us to quantitatively determine the number of sheep, horse or human erythrocytes remaining as well as the number of bacterial cells present after 24-h co-incubations in TSBD medium. Flow cytometry analyses of samples obtained from *A*. *baumannii* ATCC 19606^T^, LUH 13000 or AYE cultures containing sheep erythrocytes showed that AYE was the only strain, of the three tested strains, to be significantly hemolytic (*P* < 0.05) to sheep erythrocytes, as compared to *E*. *coli* MG1655, which was used as a hemolysis-negative control (Fig 2C). A comparison of the mean amounts of sheep erythrocytes remaining after 24-h incubations with ATCC 19606^T^, LUH 13000 or AYE under iron-chelation demonstrated a 5%, 3% and 17% reduction in sheep erythrocytes, as compared to the *E*. *coli* MG1655 hemolysis-negative control, respectively. In contrast, flow cytometry analyses showed that ATCC 19606^T^, LUH 13000 and AYE were hemolytic to human erythrocytes as demonstrated by 41%, 23% and 41% reductions in the numbers of intact human erythrocytes, respectively, with only the hemolysis caused by ATCC 19606^T^ and AYE being statistically different from the hemolysis-negative *E*. *coli* control (*P* < 0.001) (Fig 2C). All three *A*. *baumannii* strains were significantly hemolytic to horse erythrocytes (*P* < 0.001) with the percentage reduction of intact horse erythrocytes ranging from 95% after incubation with ATCC 19606^T^ or LUH 13000 to 98% after incubation with AYE. Since *A*. *baumannii* was the most hemolytic to horse erythrocytes when grown in iron-chelated TSBD, horse erythrocytes were chosen to test the effects of iron-repletion on *A*. *baumannii* mediated hemolysis. The data demonstrated a significant decrease in the hemolytic activity of ATCC 19606^T^ (*P* < 0.001), LUH 13000 (*P* < 0.01) and AYE (*P* < 0.01) when TSBD was repleted with free inorganic iron (Fig 2C). Together, flow cytometry analyses of the hemolytic activity of the three tested strains indicate that *A*. *baumannii* is poorly hemolytic to sheep erythrocytes, intermediately hemolytic to human erythrocytes and almost completely hemolytic to horse erythrocytes (Fig 2C). This increasing hemolytic activity showed a direct correlation with 0.27, 0.99 and 3.43 phosphatidylcholine/phosphatidylethanolamine ratios for sheep, human and horse erythrocytes, respectively. Taken together, the data show that the extracellular iron concentration as well as the erythrocyte phosphatidylcholine content are critical factors in the detection of *A*. *baumannii* hemolytic activity.

### *A*. *baumannii* harbors two phospholipase C genes and produces phosphatidylcholine-specific phospholipase activity

The direct correlation between the amount of phosphatidylcholine in the erythrocyte membrane shown above and the extent of hemolysis after incubation with *A*. *baumannii* suggests the potential role of a phosphatidylcholine-specific phospholipase C as the hemolytic effector. Analysis of the ATCC 19606^T^ genome available through the Broad Institute website (http://www.broadinstitute.org/) showed that this strain has two genes predicted to code for phosphocholine-specific phospholipase C enzymes. One of them (annotated as HMPREF0010_03297 and referred to as *plc1*) has a 2169-nt open reading frame (ORF) coding for a potential 722-amino acid protein (Fig 1A), which is located downstream of a gene transcribed in the same direction and predicted to code for an RNase PH. A gene coding for a putative nicotinate-nucleotide diphosphorylase is located downstream of *plc1* and transcribed in the opposite direction. The *plc1* gene corresponds to the *plc* ortholog reported as A1S_0043 in ATCC 17978, the expression of which is enhanced by 2.5-fold when bacteria are cultured in the presence of ethanol [19]. The other ATCC 19606^T^ phosphocholine-specific phospholipase C gene (annotated as HMPREF0010_00294 and referred to as *plc2*) encompasses a predicted 2229-nt ORF coding for a 742- amino acid protein (Fig 1B). This gene corresponds to the ATCC 17978 A1S_2055 gene identified by Camarena *et al*. [19]. A 48-nt intergenic region containing an inverted repeat resembling a Rho-independent transcription termination sequence follows *plc2* and separates this coding region from a potential bicistronic operon, containing a thioesterase and a lactaldehyde reductase coding region, which is transcribed in the opposite direction of *plc2*. Based on ATCC 17978 genomic data [49], a 480-nt intergenic region separates *plc2* from a predicted gene transcribed in the same direction and coding for the DNA polymerase III tau and gamma subunits. These observations indicate that the ATCC 19606^T^ *plc1* and *plc2* are coded for by monocistronic operons as it was reported for ATCC 17978 [19]. Other *A*. *baumannii* genomes including AB0057 [50], ACICU [51], ATCC 17978 [49] and AYE [22] show similar gene arrangements for the chromosomal regions harboring *plc1* and *plc2*, an observation that suggests the conservation of this genomic region across different *A*. *baumannii* isolates.

**Fig 3:**
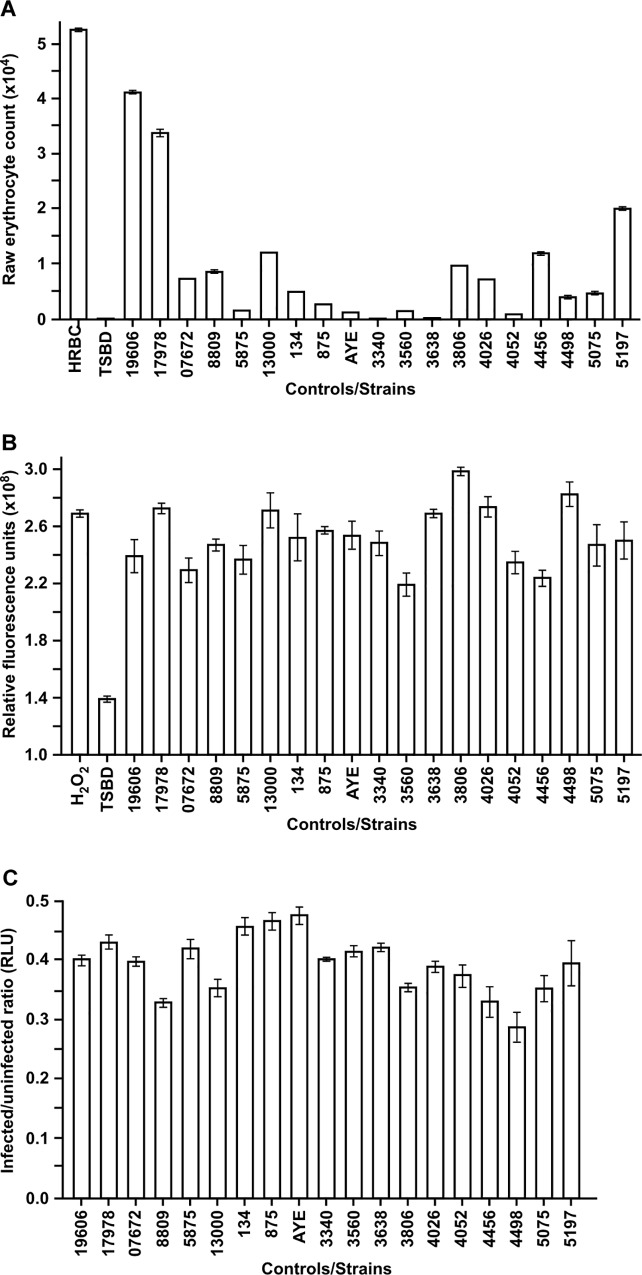
Hemolytic, PC-PLC and cytolytic activity among *A*. *baumannii* isolates. (A) Hemolytic activity was determined by the raw number of intact horse erythrocytes remaining after incubation with cells of each of the 19 *A*. *baumannii* strains. All incubations were conducted for 24 h at 37°C with shaking at 200 rpm. (B) PC-PLC activity present in TSBD culture supernatants of each strain was tested using the Amplex Red PC-PLC assay kit, using hydrogen peroxide or uninoculated TSBD medium as positive and negative controls, respectively. (C) Cytolytic activity of *A*. *baumannii* strains against A549 cells was determined with CellTiter-Glo luminescent cell viability assays. Results are expressed as the relative number of intact A549 cells remaining after incubation in the presence and absence of *A*. *baumannii* bacteria for 24 h at 37°C with 5% CO_2_. Error bars in all panels represent the standard error (SE) of the mean. Tested strains are identified as follows: 19606 and 17978 represent ATCC strains, 07672–13000 represent LUH strains, 134 and 875 represent RUH strains, and 3340–5197 represent AB wound isolates as listed in Table 1.

Currently, it is unknown if other nosocomial *A*. *baumannii* strains that have yet to be sequenced possess *plc1* and *plc2*; therefore, the presence of these genes in additional *A*. *baumannii* strains that had not yet been sequenced was tested by PCR using total genomic DNA and primers which hybridize within the respective coding regions of both phospholipase C genes (Fig 1). Amplicons of the predicted sizes, 993 bp for *plc1* and 1,167 bp for *plc2*, were obtained for all 19 tested isolates (S2 Fig) confirming the presence of these genes in a variety of *A*. *baumannii* strains. This screening study was further complemented by testing the expression of hemolytic, PC-PLC and cytolytic activities in the same 19 *A*. *baumannii* strains. Hemolysis assays using horse erythrocytes showed that all these strains had significant (*P* < 0.001) hemolytic activity as a compared to the negative control (Fig 3A). The Amplex Red PC-PLC tests showed that the PC-PLC activity of TSBD culture supernatants of all 19 strains was significantly higher (*P* < 0.001) than the negative control (Fig 3B). Finally, the CellTiter-Glo luminescent cell viability assays showed that all strains displayed cytolytic activity against A549 human alveolar epithelial cells (Fig 3C). Interestingly, HPLC analysis of lipids extracted from these cells showed a phosphatidylcholine/phosphatidylethanolamine of 2.58, which is significantly higher than the 0.27 and 0.99 ratios detected in sheep and human erythrocytes, respectively, but lower than the 3.43 value detected in horse red blood cells. It is of note that the tested strains displayed significant variations in hemolytic activity, with the ATCC 19606^T^ and AB3340 isolates producing the lowest and highest activities, respectively (Fig 3A); PC-PLC activity, with the AB3560 and AB3806 isolates producing the lowest and highest activities, respectively (Fig 3B); as well as cytolytic activity, with the AB4498 and AYE isolates being the most and least cytotoxic strains, respectively (Fig 3C).

All these data also indicate that there is no correlation between the hemolytic, PC-PLC and A549 cytolytic activities expressed by the tested strains. For example, strains ATCC 19606^T^ and ATCC 17978, which are considered non-contemporaneous and antibiotic sensitive clinical isolates displayed less hemolytic activity than most of the tested strains, several of which are modern multidrug resistant isolates. However, the PC-PLC and cytolytic activities of ATCC 19606^T^ and ATCC 17978 are comparable to that of most of the remaining tested strains. The comparative analysis of the AB3340-AB5197 strains, which were isolated from different infection sites of wounded soldiers and include the AB5075 strain considered a highly virulent strain [52], show that although there are significant variations in their hemolytic activity, PC-PLC and cytolytic activities among them are comparable to those detected in the non-military strains shown in Fig 3. Finally, the comparative analysis of the ATCC 19606^T^, ATCC 17978 and the AB5075 isolates, with the two ATCC strains being considered less virulent than the latter military isolate when tested using the *G*. *mellonella* model [52, 53], shows that although there is a correlation between their relative virulence and cognate hemolytic phenotype, such a correlation does not exist when their PC-PLC and cytolytic activities are compared. Taken together, these observations indicate that although PC-PLC seems to play a role in virulence, it is most likely that this is one of several bacterial factors responsible for the virulence of this pathogen.

**Fig 4:**
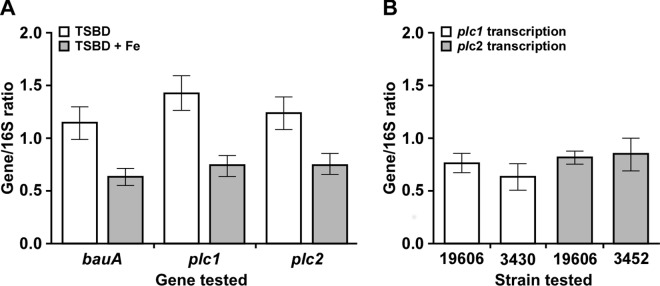
Analyses of *plc1* and *plc2* transcription in ATCC 19606^T^ and isogenic derivatives. (A) Transcriptional analyses of *plc1* and *plc2* in ATCC 19606^T^ cells grown in TSBD or TSBD supplemented with 50 μM FeCl_3_ (TSBD + Fe). Expression of *bauA* was used as a positive control for iron-regulated gene expression. (B) Transcriptional analyses of *plc1* and *plc2* genes to determine any compensatory regulation in cells of the ATCC 19606^T^ parental strain or the isogenic derivatives 3430 (*plc2*::*aph*) or 3452 (*plc1*::*aph-FRT*) cells grown in TSBD for 24 h at 37°C with shaking at 200 rpm. Expression of *plc* genes was normalized to the expression of the 16S gene, which is constitutively expressed under iron-rich and iron-chelated conditions. Error bars represent the standard error (SE) of the mean.

Preliminary assays showed that phospholipase activity was detected only when ATCC 19606^T^ bacteria were cultured in TSBD, a liquid medium that was dialyzed against Chelex 100, an insoluble polymer that binds several metals, including iron. This observation suggested that the expression of the genes responsible for the production of phospholipase activity could be iron-regulated. Quantitative RT-PCR analyses using total RNA extracted from ATCC 19606^T^ cells grown in iron-chelated or iron-repleted TSBD showed that transcription of *plc1* and *plc2* is indeed significantly higher (*P <* 0.01 and *P* < 0.05, respectively) in bacteria cultured under iron-depleted conditions when compared with TSBD supplemented with inorganic iron (Fig 4A). The approximately 0.94-fold and 0.64-fold increase in the transcription of *plc1* and *plc2* when ATCC 19606^T^ was grown in iron-chelated TSBD *vs*. iron-repleted TSBD may seem modest; however, since the product of *plc1* and *plc2* are enzymatic in nature these changes could have pronounced biological significance. These analyses also showed that the iron-regulated expression of *plc1* and *plc2* is similar to that of *bauA*, which is only increased about 0.82 fold in iron-chelated TSBD *vs*. iron-repleted TSBD and codes for the production of the BauA acinetobactin outer membrane receptor protein, which is the product of a proven iron-regulated gene [54]. The observation that the addition of FeCl_3_ to TSBD reduced the transcription of *plc1* and *plc2* to levels similar to those detected for *bauA* strongly indicate that iron indeed plays a critical regulatory role in the differential transcription of these genes (Fig 4A).

**Fig 5:**
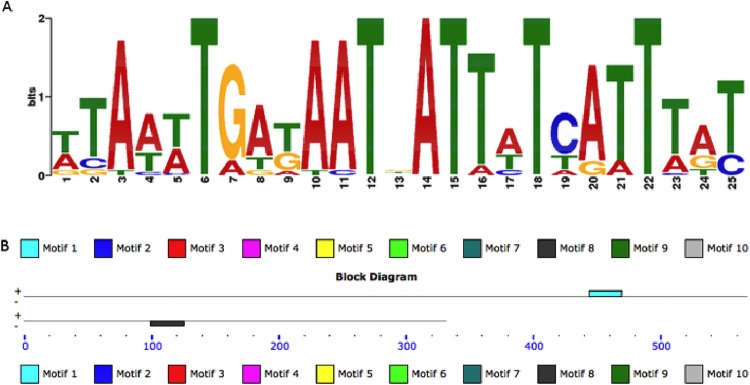
*In silico* ferric uptake repressor (Fur)-binding site prediction. The most significant prediction of the Fur-binding motif (A) and the locations of the motifs in the mRNA leader sequences of *plc1* (top line) and *plc2* (bottom line) from ATCC 19606^T^ (B). The location of the predicted Fur boxes in *plc1* (top line) and *plc2* are shown as aqua and black rectangles, respectively.

It appears likely that the iron-regulated expression of *plc1* and *plc2* is due to the presence of putative Fur-binding sites (Fig 5), which were located approximately 100 nt and 200 nt upstream of *plc1* and *plc2*, respectively, and found to be significantly related (e-value > 0.05) to the *A*. *baumannii* Fur motif recently reported [32]. This possibility is strongly supported by the observation that hemolysin and siderophore production in *V*. *cholerae* is co-regulated by iron through a Fur-dependent transcriptional regulatory process [9].

The possibility of compensatory expression between *plc1* and *plc2* was examined using ATCC 19606^T^ isogenic derivatives harboring the appropriate *plc* mutation. Quantitative RT-PCR analyses of *plc1* transcription in 3430 *plc2*::*aph* cells or *plc2* transcription in 3452 *plc1*::*aph-FRT* cells, all grown in TSBD, showed that there is not a regulatory mechanism by which *plc1* transcription compensates for the lack of *plc2* transcription or *vice versa* (Fig 4B).

Taken together, these results show that the genomes of multiple *A*. *baumannii* strains contain two phosphatidylcholine-specific phospholipase C genes, the presence of which could correlate with their capacity to express hemolytic activity, preferentially toward human and horse erythrocytes. Furthermore, the production of this activity depends on the effect of free iron on the differential transcription of *plc1* and *plc2*, which are expressed independently of each other at higher rates under iron limiting conditions.

### The *A*. *baumannii* PLC1 and PLC2 have diverged from a common ancestor protein

**Fig 6:**
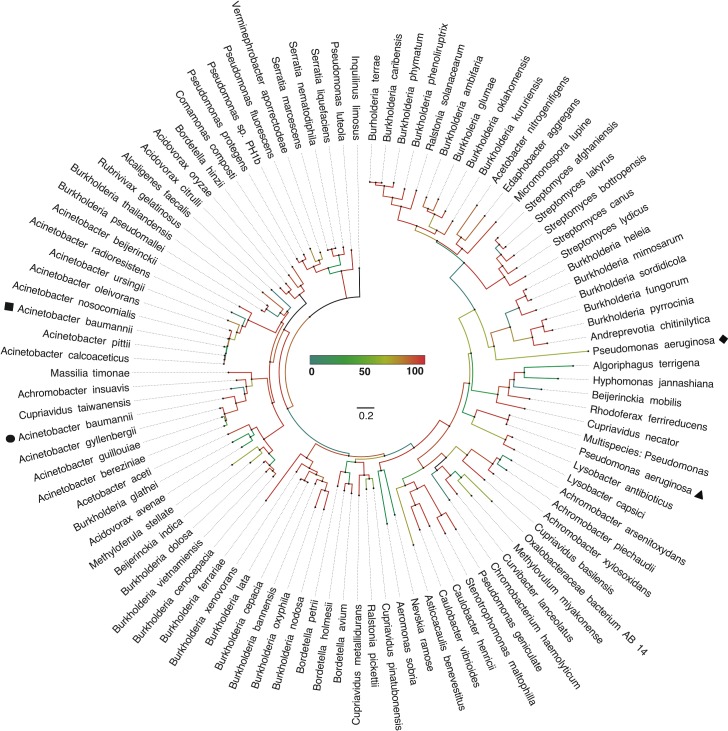
Phylogenetic analysis of phospholipase C protein sequences of *A*. *baumannii* ATCC 19606^T^. An unrooted approximate-maximum-likelihood tree inferred in FastTree showing the locations of both *A*. *baumannii* (PLC1, black square; PLC2, black circle,) and *P*. *aeruginosa* (non-hemolytic, black triangle; hemolytic, black diamond,) phospholipase C proteins relative to other PLC proteins. Percent confidence through 1,000 iterations is represented in the center of the image as a heat map in addition to a scale bar representing substitutions per site. Notably, *plc1* clusters with phospholipases coded for by other human pathogens, while *plc2* clusters mostly with phospholipases encoded by environmental isolates, some of which are invertebrate symbiotes.

Both PLC1 and PLC2 from ATCC 19606^T^ cluster with phospholipase C proteins from other known pathogens (Fig 6). PLC1 is located within a clade that includes proteins produced by seven different *Acinetobacter* species with two of them, *oleivorans* and *radioresistens* not being commonly associated with human infections. Interestingly, two of these seven species have been reported as being hemolytic, *A*. *beijerinckii* sp. nov. [55] and the *A*. *calcoaceticus* strain 1318/69 that was isolated from the urine of a 70-year old male patient [56, 57]. Our preliminary observations also indicate that *A*. *nosocomialis* M2 expresses hemolytic activity (data not shown). The hemolytic activity of *A*. *nosocomialis* M2 is not surprising since it is so closely related to *A*. *baumannii* that it was originally identified as *A*. *baumannii* M2 [58]. PLC2 groups with a more diverse clade that includes *Achromobacter*, *Cupriavidus* and *Acinetobacter* sequences, with *A*. *gyllenbergii* being reported to lyse both horse and sheep erythrocytes [55]. Interestingly, PLC2 is also in the same clade as phospholipase C produced by bacteria belonging to different genera and species. Although most of these bacteria are non-pathogenic environmental microorganisms that share symbiotic relationships with invertebrates such as *Verminephrobacter aporrectodeae* [59], some of them have been isolated from human patients such as *Massilia timonae* [60], *Bordetella hinzii* [61] and the Melioidosis agent *Burkholderia pseudomallei* [62].

More distant branches from PLC1 and PLC2 contain clusters encompassing phospholipase C proteins from *P*. *aeruginosa*. The non-hemolytic PLCN of *P*. *aeruginosa* resides in a group containing *Lysobacter antibioticus* and *Lysobacter capsici*, which play roles in the rhizospheres of rice or peppers, respectively [63, 64]. The hemolytic PLCH of *P*. *aeruginosa* groups outside a cluster of environmental isolates with *Burkholderia* spp. reported to be involved with wound infections, bacteremia and hemolysis [65–68].

### Effect of *plc* interruption on phospholipase activity and cytolysis

**Fig 7:**
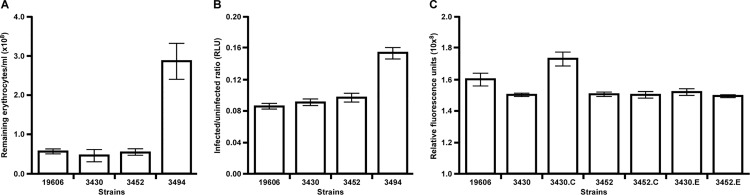
Cytolytic activity of ATCC 19606^T^ and the 3430, 3452 and 3494 isogenic derivatives. (A) Number of remaining horse erythrocytes after incubation with ATCC 19606^T^ or the isogenic derivatives 3430 (*plc2*::*aph*), 3452 (*plc1*::*aph-FRT*) or 3494 (*plc1*::*ermAM/plc2*::*aph*) in TSBD for 24 h at 37°C with shaking at 200 rpm. (B) Relative number of intact A549 cells remaining after incubation in the presence of bacteria of the ATCC 19606^T^ parental strain or the isogenic derivatives 3430, 3452 or 3494 for 24 h at 37°C in the presence of 5% CO_2_. Relative luminescence units (RLU) were determined as the ratio between the number of A549 cells present in uninfected samples and each sample infected with a different bacterial strain. (C) PC-PLC activity of TSBD culture supernatants from the ATCC 19606^T^, 3430, 3430.C, 3452, 3452.C, 3430.E and 3452.E strains. Error bars represent the standard error (SE) of the mean.

The role of the ATCC 19606^T^ PLC1 and PLC2 proteins in the lysis of erythrocytes and human epithelial cells was tested using the 3452 (*plc1*::*aph-FRT*), 3430 (*plc2*::*aph*) and 3494 (*plc1*::*ermAM/plc2*::*aph*) isogenic insertion derivatives. Interruptions in one or both of these genes did not affect the growth of these derivatives; their growth kinetics were not statistically different from that of parental ATCC 19606^T^ when cultured in TSBD under non-selective conditions (S3 Fig). Flow cytometry analyses showed that the number of intact horse erythrocytes remaining after incubation with the 3430 or 3452 isogenic derivative is not significantly different from the number of erythrocytes remaining after incubation with the ATCC 19606^T^ parental strain (Fig 7A). However, the number of erythrocytes remaining following incubation with the 3494 isogenic derivative, which has interruptions in both *plc1* and *plc2*, is more than 3-fold higher (*P* < 0.001) than the number of erythrocytes remaining after incubation with either ATCC 19606^T^ or the 3430 or 3452 isogenic derivatives (Fig 7A).

The decreased ability of the 3494 isogenic derivative to lyse erythrocytes correlates with that observed when these strains were incubated with A549 human alveolar epithelial cells. In these experiments, the number of A549 cells remaining after 24 h incubation in the presence of ATCC 19606^T^, 3430 or 3452 were not significantly different from one another; however, the number of remaining A549 cells following incubation with 3494 was significantly higher (*P* < 0.001) than the number remaining after incubation with ATCC 19606^T^, 3430 or 3452 (Fig 7B).

The role of *plc1* and *plc2* in the production of PC-PLC activity was further tested using the 3452.C (pMU1079) and 3430.C (pMU1080) derivatives, which harbor plasmid copies of the *plc1* and *plc2* parental alleles, respectively. As expected from data described above, the Amplex Red assays demonstrated that both 3452 (*P* < 0.05) and 3430 (*P* < 0.05) are significantly reduced in their ability to degrade phosphatidylcholine as compared to the parental ATCC 19606^T^ strain (Fig 7C). This figure also shows that the transformation of 3430 with pMU1080, which harbors the *plc2* coding region under the control of the pWH1266 tetracycline resistance promoter, not only restored, but also significantly enhanced the production of PLC2 (*P* < 0.01), with the latter effect being most likely due to a gene dosage effect. Unfortunately, several attempts to complement the 3452 mutant with the *plc1* parental allele (3452.C) did not restore the phospholipase activity of this derivative possibly because of regulatory mechanisms due to the fact that pMU1079 was made by cloning only the *plc1* coding region without any upstream sequences that could affect its expression in this recombinant derivative. The phospholipase activity of the 3452 and 3430 strains containing the empty *A*. *baumannii-E*. *coli* shuttle vector pWH1266 (3452.E and 3430.E, respectively) were not significantly different from that of the 3452 or 3430 strains demonstrating the pWH1266 shuttle vector used to clone the parental *plc1* and *plc2* alleles does not confer a phospholipase phenotype upon these strains. Taken together, these observations indicate that the activity of the phospholipase C proteins produced by ATCC 19606^T^ are not host cell specific and have cytolytic activity against different cell types this pathogen could encounter during infection.

### Role of *plc1* and *plc2* in virulence

**Fig 8:**
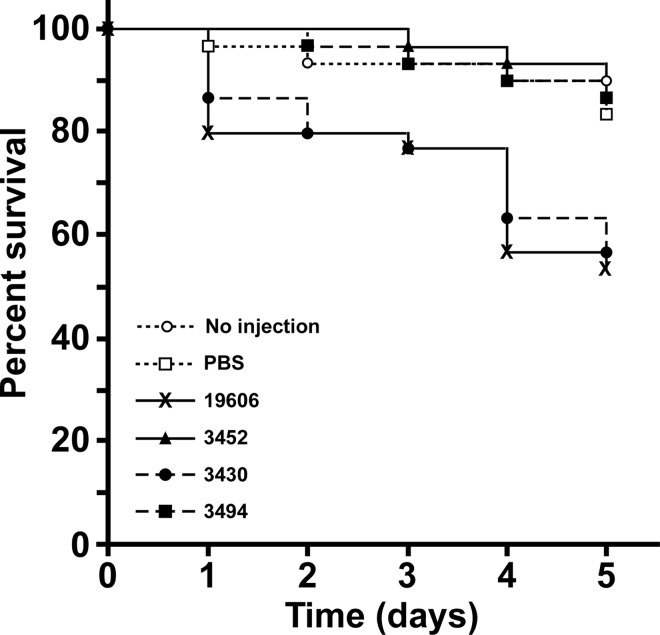
Role of *plc* in the virulence of ATCC 19606^T^. *G*. *mellonella* larva (n = 30) were injected with 1 x 10^5^ cells of the ATCC 19606^T^ parental strain or the isogenic derivatives 3430 (*plc2*::*aph*), 3452 (*plc1*::*aph-FRT*) or 3494 (*plc1*::*ermAM/plc2*::*aph*) and incubated at 37°C in darkness. Negative controls included uninjected larva or larva injected with sterile PBS. Larva survival was monitored daily for five days with removal of dead larva at times of inspection. This model showed that PLC1 but not PLC2 is critical for the virulence of the ATCC 19606^T^ strain.

The same strains used to test cytolytic activity were also used to examine the role of *plc1* and *plc2* in the virulence of *A*. *baumannii* ATCC 19606^T^ with the *G*. *mellonella* experimental virulence model we have used previously to determine the virulence role of the acinetobactin-mediated iron acquisition system [47]. Fig 8 shows that infection of caterpillars with ATCC 19606^T^ resulted in a 47% mortality rate, which is significantly higher than the 16% rate scored (*P* < 0.05) with animals that were not injected or injected with sterile PBS as negative controls. Interestingly, HPLC analysis of lipids extracted from larvae homogenates showed that the phosphatidylcholine/phosphatidylethanolamine ratio in this insect is 1.49, a value that is between the 0.99 and 2.58 values determined for human erythrocytes and A549 cells and much higher that the 0.27 ratio detected in sheep erythrocytes. There was also a significant difference in percent survival when larvae infected with ATCC 19606^T^ were compared with larvae infected with either the *plc1*::*aph-FRT* 3452 or the *plc1*::*ermAM/plc2*::*aph* 3494 double mutant (13% mortality, *P* < 0.01). Furthermore, the killing rates of these two mutants were not significantly different from each other as well as from the rates scored with animals that were not injected or injected with sterile PBS. In contrast, the death rates of caterpillars infected with the *plc2*::*aph* 3430 isogenic derivative, which actively expresses *plc1*, were very similar to those recorded after infection with the ATCC 19606^T^ parental strain (43% vs. 47%). These observations together with those collected with the cytolytic assays described above indicate that while PLC1 and PLC2 seem to play similar roles in lysing different host cells when tested either under laboratory or *ex vivo* conditions, PLC1 appears to play a more critical role during the infection of a host that mounts an innate immune response that resembles that of vertebrate animals [69].

## Discussion

*Acinetobacter baumannii* has been generally considered a non-hemolytic pathogen because, according to our observations, the detection of such an activity depends on two critical factors. One of these factors is the type of erythrocytes used in the detection tests, which are normally conducted using Columbia agar plates containing 5% sheep red blood cells. Our work demonstrates that *A*. *baumannii* is poorly hemolytic to sheep and increasingly hemolytic to human and horse erythrocytes (Fig 2C). These observations, which resemble those previously reported for the strains ACICU, AYE, ATCC 17978 and SDF using sheep and horse erythrocytes [21], are in agreement with the increasing phosphatidylcholine content we detected in these erythrocytes. Furthermore, the positive correlation between the *A*. *baumannii* hemolytic activity and the erythrocyte phosphatidylcholine content is reminiscent of a phospholipase C homolog in *P*. *aeruginosa*, where a hemolytic phospholipase C (PLCH) acts exclusively on phosphatidylcholine and sphingomyelin [70].

The effect of the co-incubation of *A*. *baumannii* with sensitive erythrocytes, such as those from horse, is apparent not only because of their lysis but also because of the generation of schistocytes due to significant cell membrane damage (Fig 2B). These fragmented red blood cells have been detected in blood smears obtained from infected neonates during an *A*. *baumannii* infection outbreak in a Saudi Arabian hospital [71]. In this case study of seven neonates, five had a total erythrocyte count lower than controls throughout the course of *A*. *baumannii* infection, and two of the seven neonates succumbed to *A*. *baumannii* bacteremia. These observations clearly underscore the potential role of hemolytic activity in the pathobiology of *A*. *baumannii*.

The second factor that determines the detection as well as the expression of *A*. *baumannii* hemolytic activity is the iron content of the culture media. Our data demonstrate that this activity is detectable when bacteria are cultured in an iron-chelated medium but not when the medium is iron rich. Columbia agar, which is used in standard clinical bacteriology methods to detect hemolytic activity, is considered a rich-nutrient medium that would have to be treated with a chelating agent such as Chelex 100 [24] to properly detect hemolytic activity. Accordingly, the differential expression of PC-PLC activity in response to iron chelation correlates well with the differential transcription of the *plc1* and *plc2* gene orthologs when Chelex 100-treated media is supplemented or not with free iron (Fig 4A), through a regulatory process that is most likely controlled by the interaction of the Fur transcriptional repressor that could bind to predicted iron boxes located upstream of these two genes (Fig 5). The iron-regulated expression of the *plc1* and *plc2* genes and the corresponding PC-PLC activity suggests that *A*. *baumannii* uses PLC1 and PLC2 for iron acquisition through the lysis of host cells and the subsequent release of iron-rich cytoplasmic contents. This possibility is further supported by the fact that iron co-regulates the expression of *plc1* and *plc2* genes as well as the expression of genes involved in the acinetobactin-mediated iron acquisition system, a response that is similar to that described in *V*. *cholerae* where the production of hemolysin and vibriobactin are regulated by a Fur-mediated process [9]. Whether *A*. *baumannii* acquires iron from intracellular pools via a siderophore-mediated system or the expression of uncharacterized hemin utilization processes [72] remains to be tested experimentally. Unfortunately, the role of Fur in the expression of the *A*. *baumannii plc* genes cannot be tested using a Fur deficient derivative since our efforts as well as that of others indicate that Fur is an essential gene. This possibility is strongly supported by a recent report showing that random mutagenesis of *A*. *baumannii* did not result in the isolation of *fur* mutants, an observation that led the authors to classify this gene as an essential genetic element in this pathogen [73].

All previous considerations and the observation that *plc1* and *plc2* are present in all sequenced *A*. *baumannii* genomes as well as the genomes of the clinical strains tested in this study, but absent in the non-pathogenic *A*. *baylyi* ADP1strain, strongly indicate a role of these phospholipases in bacterial virulence. Until this report, only *plc1* and its role in cytolysis had been tested experimentally where an *A*. *baumannii* ATCC 17978 *plc1*::*aph* isogenic insertion derivative was less effective in damaging FaDu hypopharyngeal carcinoma epithelial cells as compared to the parental strain [19]. Our data not only support the virulence role of *A*. *baumannii* PC-PLC as established by this previous report [19], but also indicate that the modest cytotoxic effect reported is most likely due to the fact that only the inactivation of both *plc1* and *plc2* results in a significant reduction of host cell damage (Fig 7). Our work also shows that the product of these two genes target different types of host cells *A*. *baumannii* could encounter during the pathogenesis of systemic infections, as well as infection of the digestive and respiratory systems as revealed by the damage this pathogen causes to erythrocytes, and the FaDu and A549 epithelial cell lines, respectively. Our data collected using laboratory and *ex-vivo* experimental conditions (Fig 7) suggest that the *plc1* and *plc2* genes code for potentially redundant cytotoxic functions. Only a double *plc1/plc2* mutant showed a significant reduction in cytolytic activity when tested using horse red blood cells and A549 human alveolar epithelial cells. However, the *G*. *mellonella* experimental infection model showed that this is not the case in an *in vivo* infection model. This model showed that PLC1 but not PLC2 is critical for the virulence of the ATCC 19606^T^ strain (Fig 8). Interestingly, *plc1* but not *plc2* proved to be transcribed at higher rates when ATCC 17978 bacteria were cultured in the presence of ethanol [19], a condition that also increases the virulence and the expression of virulence-associated traits including biofilm biogenesis and bacterial surface motility [74]. Based on all these observations, it is possible to speculate that PLC1 and PLC2 play different roles during the pathogenesis of *A*. *baumannii* infections, with PLC1 being coded for by a gene the expression of which appears to be regulated at least by iron and stress signals. These signals are critical for the virulence of this pathogen [47, 74] when tested in an invertebrate host that mounts a complex defense response that mimics that of the human host [69]. It is also possible that the biological role of these two enzymes depends on the nature of potential targets, which may reflect significant differences in phospholipid and fatty acid composition between insect and mammalian cells [75]. Interestingly, the phylogenetic analysis of PLC2 shows that this protein clusters with a phospholipase C protein from the invertebrate endosymbiote *Verminephrobacter aporrectodeae* [59] (Fig 6). This finding could explain the lackluster role of PLC2 in virulence using the invertebrate *G*. *mellonella* virulence model due to a host adaptation process. The phylogenetic analysis also showed that although many of the amino acid sequences used to construct the phylogenetic tree shown in Fig 6 are from environmental microorganisms isolated from soil, aquatic environments, or industrial sites, it is apparent that there is also a strong correlation of the bacteria producing these enzymes with plants in either antagonistic or synergistic ways (*e*.*g*., pathogenesis of blight disease in some plants or nitrogen-fixing bacteria present in the rhizosphere).

Taking into account our experimental data together with the observations published by other investigators using different clinical isolates [11, 19–21], it is apparent that *A*. *baumannii* produces two PC-PLC and three PLD phospholipases. In the particular case of the ATCC 19606^T^ strain, the three PLD phospholipases are not essential for the utilization of phosphatidylcholine as a carbon and energy source. This finding may indicate that the two PC-PLC produced by this strain could be responsible for the utilization of phosphatidylcholine as a nutrient source by the triple PLD deficient derivative [11]. It is also apparent that the ATCC 19606^T^ PC-PLC and PLD enzymes play a virulence role; although they may function differently during the infection process. The report by Stahl et *al*. [11] shows that all single and double PLD mutants display a virulence phenotype indistinguishable from the parental ATCC 19606^T^ strain and only the triple Δ*pld1-3* mutant showed a significant reduction in the killing rates throughout the course of the experiment when compared to the parental strain. However, the killing rate of this triple PLD mutant (74% for day 4 after infection) seems to be high if one considers data published by other investigators who have used the same experimental model and included in their Kaplan-Meier plots the control data collected with non-injected animals or animals injected with the same volume of sterile PBS, which unfortunately were not shown in this report [11]. Furthermore, the lack of information regarding the CFUs injected per larva rather than OD_600_ also impairs the proper comparison of our results with those recently reported using the same strain and comparable experimental conditions. Nevertheless, it is possible to speculate that there is a residual virulence activity in the triple Δ*pld1-3* ATCC 19606^T^ mutant that could account for the activity of the PLC1 but not PLC2 as we describe in this report. This critical issue as well as the question of whether the *A*. *baumannii* PLD enzymes are differentially produced in response to extracellular signals and display selective cytolytic activity in response to differences in membrane lipid composition are critical topics that remain to be tested experimentally using the proper ATCC 19606^T^ isogenic derivatives. Such knowledge will not only further our understanding of the role(s) of phospholipases in the pathobiology of *A*. *baumannii*, but also provide critical information needed to determine whether these enzymes could be used as alternative targets to treat the severe infections caused by this pathogen, particularly by emerging multi-drug resistant isolates.

## Supporting Information

S1 Fig Sheep erythrocytes incubated in the presence of ATCC 19606^T^ bacteria. DIC image of sheep erythrocytes incubated in TSBD inoculated with ATCC 19606^T^. The scale bar is equal to 10 μm.
(TIF)

S2 Fig Detection of *plc1* and *plc2* in the genomes of *A*. *baumannii* strains. Agarose gel electrophoresis of internal amplicons of *plc1* (A) or *plc2* (B) using total genomic DNA isolated from 19 *A*. *baumannii* strains and primers 3824 and 3826 or 3822 and 3827 (Table 1 and Fig 1), which hybridize internally to *plc1* or *plc2*, respectively. MWM, *Hin*dIII-digested λ DNA.
(TIF)

S3 Fig Growth of the ATCC 19606^T^ parental strain and the 3430, 3452 and 3494 isogenic derivatives. The OD_600_ values of each strain grown in TSBD at 37°C for 24 h with shaking at 200 rpm were determined hourly. Error bars represent the standard error (SE) of the mean.
(TIF)

S1 Table Primers Used in This Work. (DOCX)
