# Supplementary figures and images for: Iron-Regulated Phospholipase C Activity Contributes to the Cytolytic Activity and Virulence of Acinetobacter baumannii
Source: PLoS One. 2016 Nov 22;11(11):e0167068. doi: 10.1371/journal.pone.0167068 (PMC5119829; doi:10.1371/journal.pone.0167068)

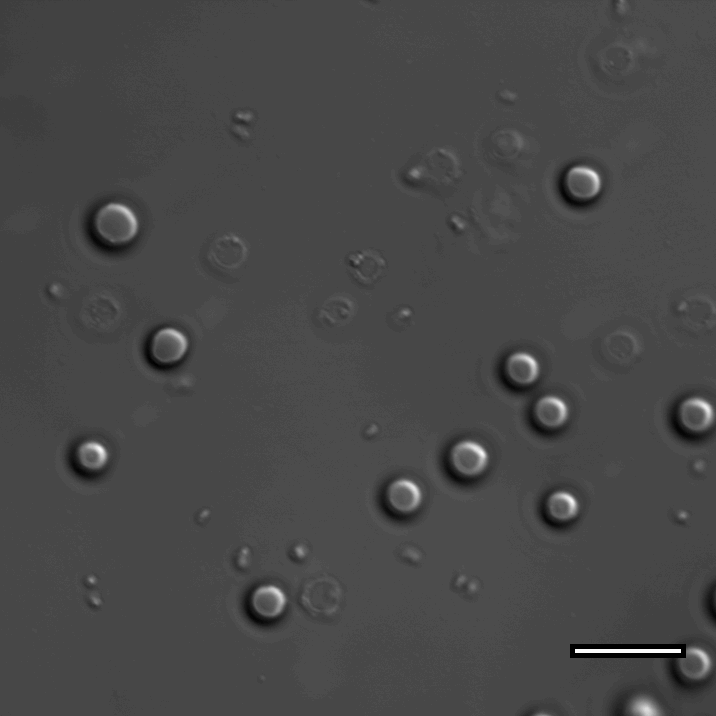

Supplement: S1 Fig — DIC image of sheep erythrocytes incubated in TSBD inoculated with ATCC 19606T. The scale bar is equal to 10 μm. (TIF) [file pone.0167068.s001.tif]

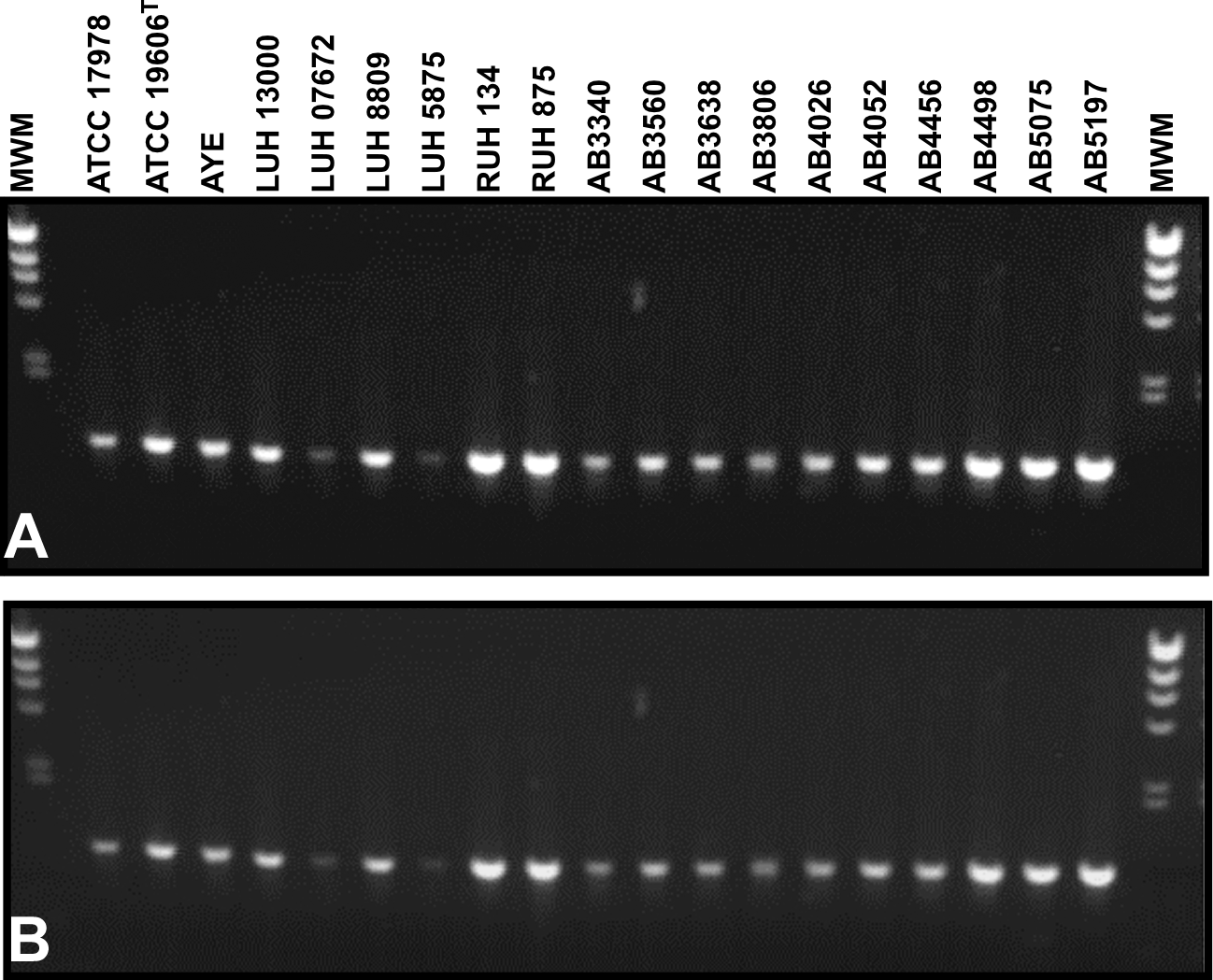

Supplement: S2 Fig — Agarose gel electrophoresis of internal amplicons of plc1 (A) or plc2 (B) using total genomic DNA isolated from 19 A. baumannii strains and primers 3824 and 3826 or 3822 and 3827 (Table 1 and Fig 1), which hybridize internally to plc1 or plc2, respectively. MWM, HindIII-digested λ DNA. (TIF) [file pone.0167068.s002.tif]

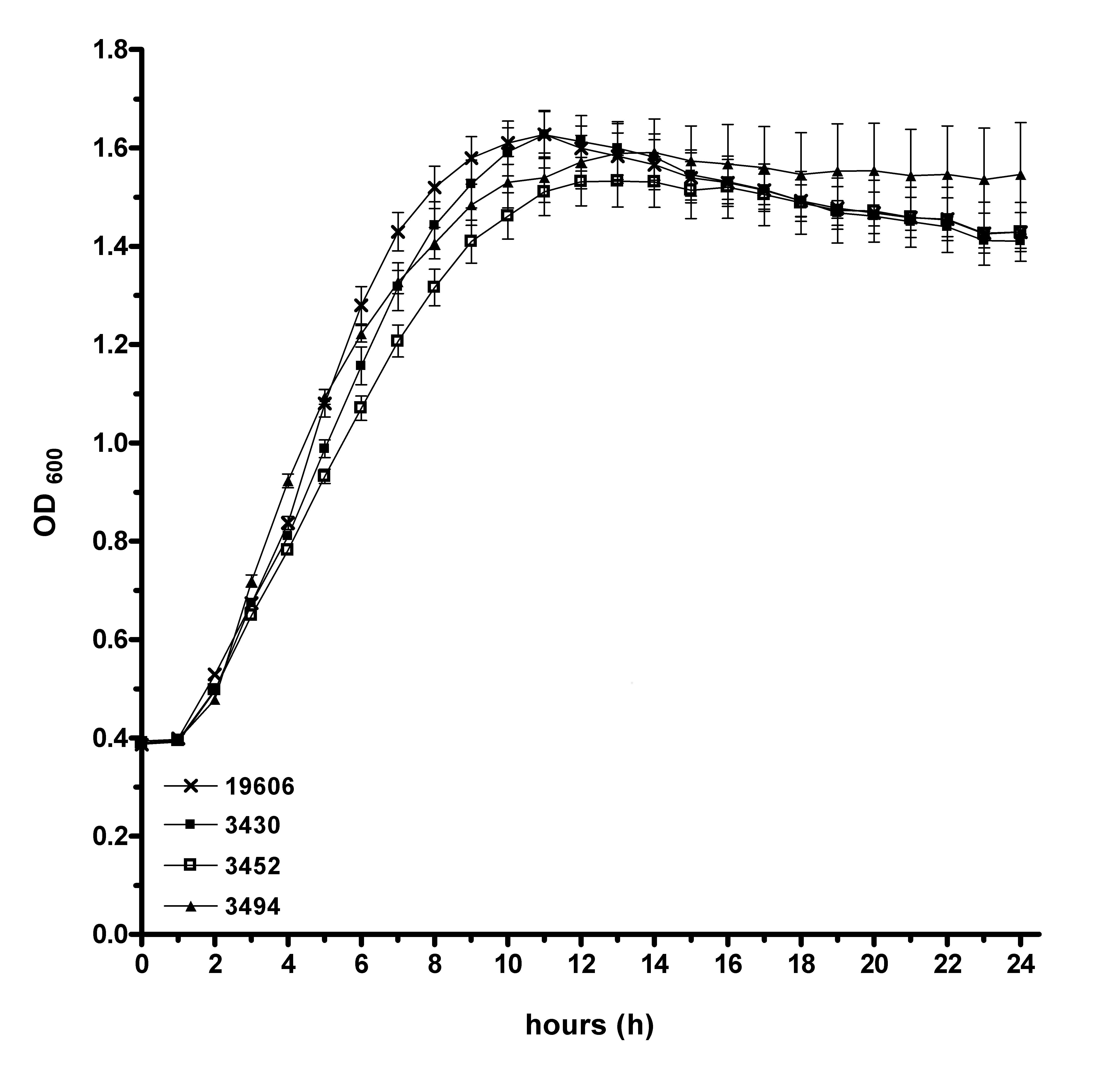

Supplement: S3 Fig — The OD600 values of each strain grown in TSBD at 37°C for 24 h with shaking at 200 rpm were determined hourly. Error bars represent the standard error (SE) of the mean. (TIF) [file pone.0167068.s003.tif]
