# Supplementary material for: Iron-Regulated Phospholipase C Activity Contributes to the Cytolytic Activity and Virulence of Acinetobacter baumannii
Source: PLoS One. 2016 Nov 22;11(11):e0167068. doi: 10.1371/journal.pone.0167068 (PMC5119829; doi:10.1371/journal.pone.0167068)
Supplement: S1 Table — (DOCX) [file pone.0167068.s004.docx]

**S1 Table. Primers Used in This Work.**

| Number | Nucleotide sequence*^a^* |
| --- | --- |
| 3171 | 5’-GTTGCTGACTCATACCAG-3’ |
| 3172 | 5’-ATTCCAACATGGATGCTG-3’ |
| 3815 | 5’-TTCCGGTACCAGAACAAA-3’ |
| 3822 | 5’-CATGATTACACGTCGTAAATT-3’ |
| 3823 | 5’-CTTAGATCATTGCGGGATCACTA-3’ |
| 3824 | 5’-ATGAATCGTCGCGAATTTCTTTT-3’ |
| 3825 | 5’-TTAAGACTCTAAATATCCCATA-3’ |
| 3826 | 5’-ATACCAAGCGCCTTGTAC-3’ |
| 3827  3892  3893  3894  3895 | 5’-GGCGATGGCATATGGTCA-3’  5’-GCGGATCCCATGATTACACGTCGTAA-3’  5’-GCGGATCCCTTAGATCATTGCGGGAT-3’  5’-GGATCCATGAATCGTCGCGAATTT-3’  5’-GCGGATCCTTAAGACTCTAAATATCCC-3’ |
| 3905 | 5’-CGACTCAGATAATTCATCG-3’ |
| 3906 | 5’-GGATGAGCTAAACCAAGC-3’ |
| 3918 | 5’-CGAAACACGCTTTGAAGC-3’ |
| 3966 | 5’-CCTAGAGATAGTGGACGTTACTCG-3’ |
| 3967 | 5’-CCAGTATCGAATGCAATTCCCAAG-3’ |
| 3970 | 5’-GGAATGTATCGAGATGGAGATGC-3’ |
| 3971 | 5’-GTTAACACCACGTGTCACGC-3’ |
| 3972 | 5’-GGAACCAATGGACCTACAGG-3’ |
| 3973 | 5’-CGGCGATATTGCTTGAAACC-3’ |
| 3974 | 5’-CGTTGTAAAGCAGATGGTAAGGTG-3’ |
| 3975 | 5’-GGTCATAACGGCCTAAGTTATCG-3’ |
| 4003 | 5’-GTGTAGGCTGGAGCTGCTTC-3’ |
| 4004 | 5’-CGCCATTAATTCACTGATCA-3’ |
| 4017 | 5’-TTAATACCAAGCGCCTTGTAC-3’ |
| 4018 | 5’-GAATACTTTAACCATCCAGCAGTAG-3’ |
| 4046 | 5’-GCAAACTTAAGAGTGTGTTG-3’ |
| 4047 | 5’-CCTTTAGTAACGTGTAACTTTC-3’ |
| *^a^*Underlined sequence indicates the addition of a stop codon | |
